# Computational Fragment-Based Binding Site Identification by Ligand Competitive Saturation

**DOI:** 10.1371/journal.pcbi.1000435

**Published:** 2009-07-10

**Authors:** Olgun Guvench, Alexander D. MacKerell

**Affiliations:** Department of Pharmaceutical Sciences, School of Pharmacy, University of Maryland Baltimore, Baltimore, Maryland, United States of America; University of California San Francisco, United States of America

## Abstract

Fragment-based drug discovery using NMR and x-ray crystallographic methods has proven utility but also non-trivial time, materials, and labor costs. Current computational fragment-based approaches circumvent these issues but suffer from limited representations of protein flexibility and solvation effects, leading to difficulties with rigorous ranking of fragment affinities. To overcome these limitations we describe an explicit solvent all-atom molecular dynamics methodology (SILCS: Site Identification by Ligand Competitive Saturation) that uses small aliphatic and aromatic molecules plus water molecules to map the affinity pattern of a protein for hydrophobic groups, aromatic groups, hydrogen bond donors, and hydrogen bond acceptors. By simultaneously incorporating ligands representative of all these functionalities, the method is an *in silico* free energy-based competition assay that generates three-dimensional probability maps of fragment binding (FragMaps) indicating favorable fragment∶protein interactions. Applied to the two-fold symmetric oncoprotein BCL-6, the SILCS method yields two-fold symmetric FragMaps that recapitulate the crystallographic binding modes of the SMRT and BCOR peptides. These FragMaps account both for important sequence and structure differences in the C-terminal halves of the two peptides and also the high mobility of the BCL-6 His116 sidechain in the peptide-binding groove. Such SILCS FragMaps can be used to qualitatively inform the design of small-molecule inhibitors or as scoring grids for high-throughput *in silico* docking that incorporate both an atomic-level description of solvation and protein flexibility.

## Author Summary

Fragment-based drug discovery is based on a simple yet powerful principle: instead of trying to screen through the vast number of possible drug-like compounds during the drug discovery process, screen representative drug-like fragments, which are far fewer in number. Once a suitable fragment is discovered, it can then be built up or linked with other fragments to give a drug-like molecule. Because such fragments are small, even “good” fragments bind weakly to their targets, therefore requiring significant time, labor, and materials costs for experimental detection and characterization of binding. In the present work, we describe a computational approach to the problem of detecting and characterizing fragment binding. Importantly, the method provides atomic-resolution results and also explicitly takes into account the effect that molecular water has on binding and the inherent flexibility of protein targets. The methodology is demonstrated by application to the BCL-6 protein, which is implicated in a variety of cancers, is conceptually easy to understand, and can yield results in a matter of days using present-day commodity computers.

## Introduction

Fragment-based drug discovery relies on a simple premise: identify small-molecule fragments that bind to a target region of the protein and then evolve or link them to create a larger high-affinity molecule. To a first approximation, the binding free-energies of fragments bound in non-overlapping poses are additive [1]. Therefore, linking two such fragments with millimolar affinities (4 kcal*mol^−1^) will yield a single molecule with micromolar affinity (8 kcal/mol), which is of sufficient affinity to serve as a “hit” for lead optimization [2]. Since the chemical space spanned by small fragments is orders of magnitude smaller than that spanned by molecules of sufficient size to be hits, it becomes feasible to screen a fragment library representative of the full extent of chemical space [3].

Nature imposes an upper limit on the contribution per ligand heavy atom to the binding free-energy [4], commonly referred to as “ligand efficiency” (LE) [5]. This limit means that even the best fragments (LE 0.4–0.5 kcal*mol^−1^ per heavy atom [3]) still have weak affinities for their targets, making their screening by traditional assays difficult. Consequently, fragment-based drug discovery relies on sensitive biophysical methods to detect fragment binding. Among these methods are NMR spectroscopy (“SAR-by-NMR”) [6] and x-ray crystallography [7]. These two methods additionally benefit from the fact that they yield structural information about fragment binding poses, which is useful for confirming that two fragments indeed bind to two adjacent sites and can be productively linked. Despite their utility, there are significant time, labor, and materials costs associated with experimental fragment-based drug discovery approaches.

Computational approaches to fragment-based drug discovery hold out the promise of mitigating the costs of experimental fragment-based drug discovery. Currently, in computational approaches the protein is assumed to be rigid and fragments sample the surface of the rigid protein using an energy function that models the solvent environment as a continuum [8]–[12]. As a result, these methods are limited in their ability to accurately account for protein conformational heterogeneity and solvation effects, contributions that are essential to compute free energies of binding [13]. In reality, proteins can accommodate ligands by undergoing conformational changes [14],[15], and water plays an important role in protein∶ligand binding affinity [16]–[18]. Significant advances have been made toward incorporating protein flexibility, for example by screening against multiple different rigid protein conformations [19]–[21], and toward more accurate modeling of solvation effects in energy functions [22]. Nonetheless, approximations used in computational approaches to date can still limit the accuracy of fragment placement and scoring, and, ultimately, the determination of the most suitable fragment for a selected region of the protein.

All-atom explicit-solvent molecular dynamics (MD) simulations of proteins give an atomic-level-of-detail description of the motions of both protein and water atoms at relevant temperature and pressure [23]. MD samples a Boltzmann distribution of thermally accessible protein conformations, and with the ability of MD to reach the nanosecond timescale, the sampled conformations can include changes in sidechain dihedral angles as well as loop motions. Furthermore, MD simulation-based methods are able to determine the absolute binding free energy of a ligand to a protein to, in the best cases, within *RT* of the experimental value [15], [24]–[32]. However, such MD free-energy calculations are computationally expensive, limiting MD simulations from being used directly for high-throughput *in silico* screening.

Toward overcoming present limitations in fragment-based computational drug design we describe a new method that combines ideas from experimental fragment-based drug discovery with all-atom explicit-solvent MD. The method (SILCS: Site Identification by Ligand Competitive Saturation) involves computationally immersing a protein in an aqueous solution simultaneously containing different types of small molecules, with each at a concentration of ∼1 M. The protein+small molecule+water system is then subjected to multiple MD simulations allowing for competitive binding of the small molecules to the protein. Snapshots from the MD trajectories are combined to generate 3D probability maps (FragMaps) that reveal what types of functionalities bind most strongly to different parts of the protein surface. Because they are generated from MD simulations, SILCS FragMaps incorporate both protein mobility, with a Boltzmann distribution of conformations, and atomic-level solvation effects, thereby yielding FragMaps that represent rigorous free energy distributions. Notably, the method requires minimal time, labor, and materials compared to experimental approaches.

As a test case, SILCS FragMaps were generated for the BTB domain of the BCL-6 oncoprotein [33],[34]. The SILCS FragMaps, from MD simulations initiated using the BCL-6 conformation in the BCL-6∶SMRT protein∶peptide cocrystal, recapitulate the pattern of aliphatic, aromatic, hydrogen bond donor, and hydrogen bond acceptor interactions seen at the BCL-6∶SMRT cocrystal interface. Additionally, these same FragMaps also recapitulate the interaction pattern seen in the BCL-6∶BCOR protein∶peptide cocrystal, which has important differences arising from sequence and structure variation in the C-terminal halves of the SMRT and BCOR peptides. Furthermore, the simulations sample the BCL-6 His116 sidechain conformation seen in the BCL-6∶BCOR cocrystal, a conformation that is required for hydrogen bonding with BCOR Ser508 and significantly different from that in the SMRT-bound BCL-6 MD starting conformation, emphasizing the ability of the presented approach to account for protein flexibility.

## Results/Discussion

The SILCS methodology is as follows: immerse the protein in a high-concentration (∼1 M) aqueous solution of multiple small molecules, run multiple nanosecond-length MD simulations of the composite protein+small molecule+water system, compute probability maps for small molecule and water binding around the protein for each simulation, and combine probability maps of the same type from all simulations to generate a single probability map (FragMap) of each fragment type. Once generated the FragMaps have the potential to be used to qualitatively inform the assembly of an inhibitor or as docking grids for high-throughput *in silico* screening. Two important aspects of the methodology to consider prior to generation and analysis of the FragMaps are the choice of small molecules and overcoming small molecule aggregation.

### Choice of small molecules

The majority of moieties on drug-like molecules that target proteins fall into four classes: aliphatic, aromatic, hydrogen bond donor, and hydrogen bond acceptor. This reflects the relatively limited chemical diversity of amino acid sidechains. Salt bridges between two amino acids are a special case of hydrogen bonding, since the interaction is never directly between two charged heavy atoms but between a negatively-charged oxygen and the proton on a positively-charged nitrogen. Fragment libraries generated from existing drugs and drug-like molecules reflect this limited diversity, being largely composed of hydrogen bond donors consisting of amides, hydrogen bond acceptors of carbonyls and ethers, hydrophobic groups of small-length aliphatic chains, and aromatic/cyclic groups of benzene [35].

The first goal in the choice of small molecules for use in this initial implementation of SILCS was to minimize the set of fragments so as to be able to maximize their individual concentrations, which in turn maximizes binding and helps convergence on the MD timescale. To this end, a minimalist small-molecule set was selected that contains hydrophobic aliphatic moieties, aromatic moieties, hydrogen bond donors, and hydrogen bond acceptors. Propane was chosen to represent hydrophobic aliphatic groups because the termini are small enough to fit into cavities only large enough to accommodate a methyl group, while the molecule itself is large enough to disrupt the hydrogen bonding structure of water so as to induce strong hydrophobic binding [36]. Additionally, unlike longer-chain alkanes, propane is essentially a rigid body excepting the rotation of the two terminal methyl groups, and thus convergence of internal degrees of freedom is not an issue. Benzene was selected to represent aromatic groups as it occurs in over 40% of drug-like compounds and is four times more common than the next most-common aromatic moiety [35]. Finally, water was used as a small molecule that contains both hydrogen bond donating and accepting capabilities. Water is at a concentration of 55 M in solution and also has no internal conformational degrees of freedom, again promoting convergence on the MD timescale. Other small-molecule possibilities for hydrogen bond donors and acceptors include acetone, formaldehyde, and small amides, but these would necessarily be at much lower concentrations than water, hindering convergence. Additionally, they contain several different functionalities, such as the methyl groups in acetone and the combined hydrogen bond donor and acceptor moieties in an amide, which can make binding analysis more difficult.

The second goal in a choice of small molecules for use in SILCS was to minimize their sizes to maximize convergence, both by facilitating reversible binding on the MD timescale and allowing for fast diffusion through the bulk solvent. Even with a high-ligand efficiency, i.e. 0.4 kcal*mol^−1^ per heavy atom, fragments consisting of 3–6 heavy atoms will have binding affinities of only 1.2 to 2.4 kcal*mol^−1^ (100 millimolar to 10 millimolar). While such weak binding affinity can be a liability in an experimental approach as it may push the limits of detection, it is an asset in the SILCS approach, allowing for ligand exchange from a binding site on the MD timescale, facilitating the implementation of a competitive *in silico* binding assay. Another benefit of molecules having only 3–6 heavy atoms is that their high diffusion rates lead to quick mixing and rapid translation to different regions of the protein surface. Thus, small molecules of minimal molecular size are beneficial both because of rapid binding exchange with the protein and rapid diffusion around the protein.

It should be emphasized that the SILCS approach is amenable to a wide range of fragment-like small molecules. The fragment molecules selected for the present study were chosen for computational expediency, as proof-of-principle, and because they represent a minimal set that includes aliphatic, aromatic, hydrogen bond donor, and hydrogen bond acceptor moieties. Larger fragments and/or fragments with a greater diversity of functional groups may prove useful in developing a more fine-grained classification of preferred functionalities beyond simply aliphatic, aromatic, hydrogen bond donor, and hydrogen bond acceptor. These may encompass different types of hydrogen bonding groups such as ethers, amides, amines, esters, and carbonyls, as well as heterocyclic aromatics and molecules with halides, to name some possibilities.

### Overcoming small-molecule aggregation

**Figure 1:**
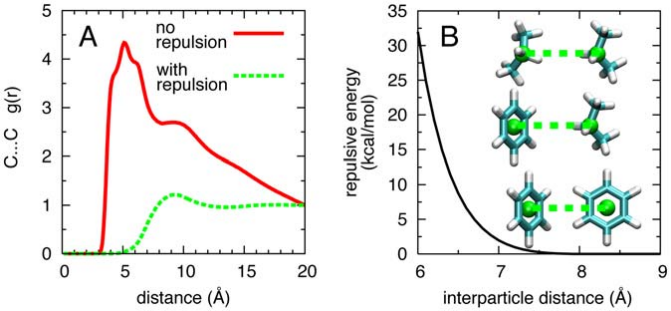
Effect of repulsive interactions between pairs of fragment molecules. (A) Carbon…carbon (C…C) radial distribution functions *g*(*r*) for an aqueous solution containing 1 M propane and 1 M benzene with and without a repulsive intermolecular interaction. (B) Location of repulsive interaction energy sites on propane and benzene molecules (spheres), and the interaction energy profile between two repulsive interaction energy sites.

To ensure binding of small low-affinity molecules, a high concentration (∼1 M) of each small molecule is used in the simulations. However, a simulation of a solution of 1 M propane and 1 M benzene in water is prone to severe hydrophobic aggregation, as seen in the intermolecular carbon…carbon (C…C) radial distribution function *g*(*r*), which traces the relative probability of observing this pair of atoms at a given separation distance. The C…C *g*(*r*) has a large peak at 5 Å (Figure 1*A*, “no repulsion”), associated with the distance between carbons in two fragments that are in direct contact. This trace slowly decays with increasing distance, reflecting the fact that in an aggregate in water, it is much more likely to have two hydrophobic fragments adjacent to each other than at a larger separation. Such aggregation drastically reduces the effective concentration of the fragments, which in turn hampers sampling of the protein surface and prevents SILCS FragMap convergence.

Because SILCS is a computational approach, it is possible to modify the interactions between hydrophobic/aromatic fragments to prevent aggregation. This can be done by introducing a repulsive interaction energy between fragments that comes into effect only when two fragments come closer than a given interaction distance. This repulsive interaction energy is only applied to selected fragment∶fragment interactions, while all fragment∶water, fragment∶protein, water∶water, water∶protein, and protein∶protein interactions remain unperturbed. For convenience, the repulsive interaction is implemented using the Lennard-Jones force field term [37] by adding an additional massless particle to the geometric center of each benzene molecule and the central carbon of each propane molecule. These particles serve as interaction sites for the inter-fragment repulsive interaction energy. Lennard-Jones parameters (*ε* = −0.01 kcal/mol; *R*_min_ = 24.0 Å) combined with a switching function [38] operating between 5 Å and 8 Å yield an energy vs. distance profile that is purely repulsive (Figure 1*B*). With this additional repulsive interaction energy in effect, even at very high concentrations the small molecules will not aggregate. Thus, in the simulation of 1 M propane and 1 M benzene in water, the *g*(*r*) contact peak at 5 Å disappears, indicating the lack of direct intermolecular C…C contacts, and the flat *g*(*r*) trace at larger distances indicates a homogeneous distribution of molecules in solution (Figure 1*A*, “with repulsion”). In principle, such a repulsive term can make hydrophobic fragments that associate with the protein surface compete unphysically with other directly adjacent hydrophobic fragments. For example, the form of the repulsive potential (Figure 1*B*) will prevent the formation of a stacked benzene dimer in a binding pocket. It will, however, allow for two benzene molecules to simultaneously bind unimpeded in two adjacent pockets on the protein surface.

### Selection of target protein

The BTB domain of the BCL-6 protein was chosen as a test case for the SILCS method because of several favorable properties. The first is that it has two-fold symmetry, with two identical symmetry-related binding sites [33], allowing for measuring convergence of fragment sampling by analyzing the two-fold symmetry in the SILCS FragMaps. A second reason is that the binding of native ligands to the two binding sites shows no cooperativity [33]; thus, the binding sites are independent of each other and the occupancy of one site will not affect the occupancy of the other. A third reason is that two known ligands for BCL-6, SMRT and BCOR, are peptides 17 amino-acids in length that bind in extended conformations to the same groove over a large contact-area [33],[34], allowing for comparison of FragMaps over a large portion of the protein. Fourth, there is thermodynamic data available from competition assays using single-residue alanine or glycine-substituted analogs of these two peptides for every position in each peptide. Fifth, SMRT and BCOR have different binding modes in the BCL-6 peptide-binding cleft and lack sequence similarity. The different binding modes include BCL-6 sidechains in the binding cleft assuming different conformations in the presence of SMRT vs. BCOR. And finally, BCL-6 has clinical importance because of its association with diffuse large B-cell lymphoma, and competitive inhibitors that bind to the BCL-6 peptide-binding cleft may have therapeutic applications.

### Convergence is achievable in the MD timescale

**Figure 2:**
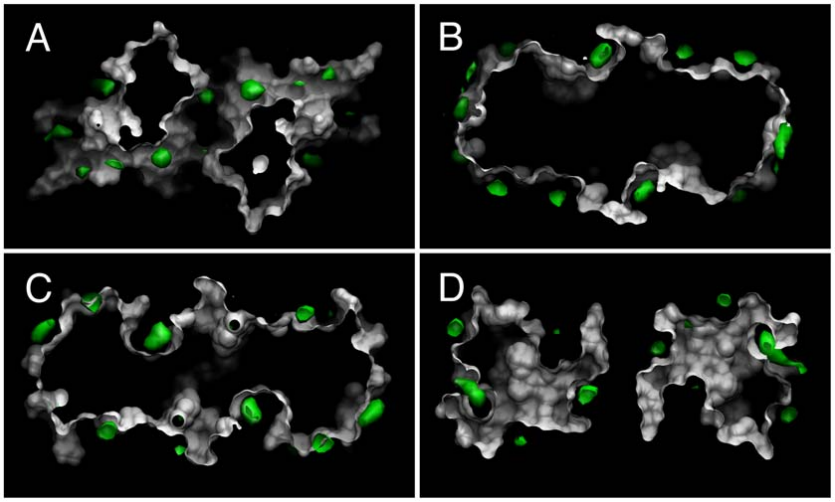
Successive slices (A–D) of the aliphatic carbon atom FragMap (green), generated by mapping propane carbon atoms, and the crystallographic BCL-6 molecular surface (white) taken perpendicular to the two-fold symmetry axis of the protein.

Convergence of the SILCS FragMaps was facilitated by the selection of propane, benzene, and water as the “fragments,” by the use of ∼1 M propane and benzene concentrations, and by combining results from 10 independent 5-ns SILCS MD simulations (see Methods). The two-fold symmetry of the BCL-6 protein with its two symmetric binding sites and non-cooperative binding allows for using two-fold symmetry in the FragMaps as a measure of convergence. Analysis of the separate 5-ns simulations shows them to yield somewhat different FragMaps that do not have exact two-fold symmetry (not shown); however, FragMaps generated as the ensemble average of all ten 5-ns simulations do exhibit the expected symmetry. To visualize the extent of convergence, slices of the aliphatic carbon atom FragMap from propane along with the protein molecular surface were taken perpendicular to the two-fold symmetry axis of the protein. These slices clearly demonstrate the expected two-fold symmetry in the FragMap, and hence convergence (Figure 2). Similarly converged results are seen for the aromatic carbon atom FragMap generated by mapping benzene carbon atoms and the hydrogen bond donor and acceptor FragMaps generated by mapping water molecules (Figures S1, S2, and S3).

**Figure 3:**
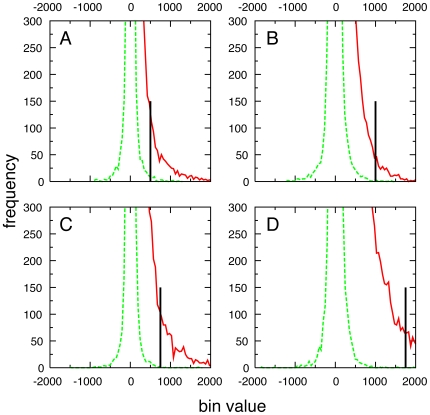
Frequency histograms of FragMap (solid red) and difference map (dashed green) bin counts for (A) aliphatic carbons, (B) aromatic carbons, (C) hydrogen bond donors, and (D) hydrogen bond acceptors. Solid black vertical lines are located at the isocontour value used for visualizing FragMaps (Figures 4–6).

To more rigorously evaluate the extent of convergence, difference maps were obtained by subtracting FragMaps based on half the MD simulation data from those based on the other half. This was done for each type of FragMap by creating one map from five 5-ns simulations, a second from the remaining five 5-ns simulations, and then subtracting the first map from the second. For fully converged results these difference maps would have bin counts of zero for all volume elements (i.e. fragment atom counts in 1 Å×1 Å×1 Å cubic volume elements as described in Methods). Presented in Figure 3 are the frequency distributions of bin counts from the FragMaps (solid red) and the difference maps (dashed green), as well as bin count cutoff values used for the visualization of isosurfaces (see below) for the four fragment types. The difference map distributions are all centered around zero as expected for random errors, while the distributions from the FragMaps are all non-negative and have much higher bin counts. The difference distributions, with the exception of the aliphatic distribution (Figure 3*A*), go to zero below the cutoff value used for visualization, demonstrating convergence between the two data sets. In the case of the aliphatic difference map, the bin count at the isovalue cutoff is only 6% of that for the actual FragMap. These results indicate that while the FragMaps are not fully converged, the extent of convergence is adequate to identify regions of high probability for the different fragment types, which is ultimately the goal of the SILCS approach. Further, the difference map analysis shows that the different sets of SILCS simulations are generating the same affinity pattern for fragment molecules. Because each of the ten SILCS simulations was started with a different random ordering of fragment molecules on a cubic grid (see Methods), the similarities between the FragMap data from the grouping into two sets of five simulations likely reflect convergence as opposed to redundant unconverged results.

### FragMaps identify key binding interactions

**Figure 4:**
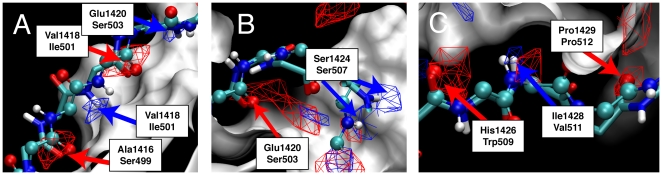
Hydrogen bond donor (blue mesh) and hydrogen bond acceptor (red mesh) SILCS FragMaps isosurfaces overlapping with the (A) N-terminal, (B) central, and (C) C-terminal residues in the SMRT and BCOR peptides. SMRT peptide atoms are represented as balls-and-sticks and BCOR atoms as tubes. The BCL-6 molecular surface from the BCL-6∶SMRT complex is also shown, and the residue/FragMap overlaps are labeled.

**Figure 5 pcbi-1000435-g005:**
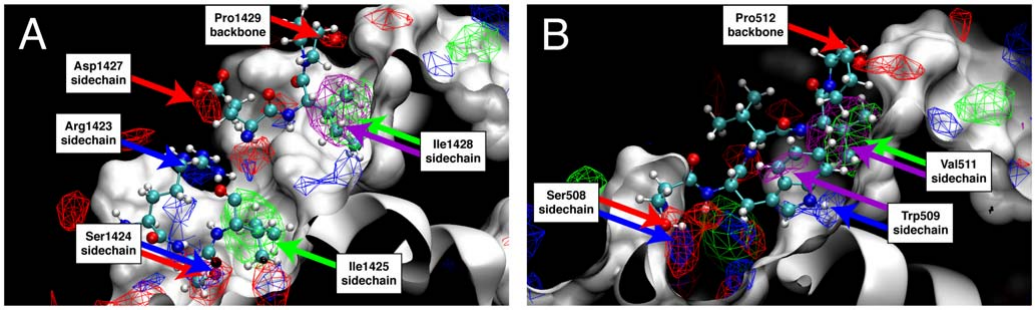
SILCS FragMaps for BCL-6 overlapping with the C-terminal residues of the SMRT and BCOR peptides. The molecular surface of the BCL-6 protein from the BCL-6∶SMRT cocrystal is shown in white. High-probability isosurfaces from the FragMaps are represented as meshes, with the aliphatic carbon FragMap in green, the aromatic carbon in purple, the hydrogen bond donor in blue, and hydrogen bond acceptor in red. Thermodynamically important residues in (A) the SMRT peptide and (B) the BCOR peptide are shown as sticks, and the residue/FragMap overlaps are labeled.

SILCS FragMaps were compared with the crystal structures of the BCL-6∶SMRT and BCL-6∶BCOR complexes to validate the method's ability to identify known binding interactions. FragMaps overlaid on the BCL-6∶SMRT and BCL-6∶BCOR structures are shown in Figures 4 and 5: Figure 4 focuses on interactions with the peptide backbones, while Figure 5 focuses on the C-terminal regions of the peptides, which contain the majority of the thermodynamically important interactions between the peptides and BCL-6 [34]. FragMap isosurfaces for hydrogen bond donors are in blue, hydrogen bond acceptors in red, aliphatic carbons in green, and aromatic carbons in purple, with the sites of discussion emphasized using arrows of the same color.

BCL-6 binding interactions conserved between the non-homologous SMRT and BCOR peptides are exclusively hydrogen bonding interactions with the peptide backbones [34], and the hydrogen bond donor and acceptor FragMaps show these conserved interactions. Starting from the N-termini of the two peptides, the backbones of SMRT Ala1416 and Val1418, and BCOR Ser499 and Ile501 act as hydrogen bond acceptors, and of SMRT Val1418 and Glu1420, and BCOR Ile501 and Ser503 as donors, all of which are recapitulated by high-probability regions in the corresponding FragMaps (Figure 4*A*). Toward the middle of the peptides, high-probability regions overlap with SMRT Glu1420 and BCOR Ser503 as hydrogen bond acceptors to BCL-6, and SMRT Ser1424 and BCOR Ser507 as donors (Figure 4*B*). Finally, at the C-termini, hydrogen-bond acceptor FragMap overlap is observed with SMRT His1426 and Pro1429 as well as BCOR Trp509 and Pro512, while hydrogen-bond donor FragMap overlap is seen for SMRT Ile1428 and BCOR Val511 (Figure 4*C*). The only peptide backbone hydrogen bonding interactions for SMRT not detected by SILCS are at the ends of the peptide, namely Ala1416 as a hydrogen bond donor and Ile1428 as a hydrogen bond acceptor, which may be explained by the high crystallographic temperature factors of these residues [33]. In the case of the BCOR peptide, only the Ser508 backbone is not detected as a strong hydrogen bond donor. Thus, in eighteen out of twenty-one cases, high probability regions in SILCS FragMaps recapitulate the location of both SMRT and BCOR peptide backbone hydrogen bonds to BCL-6.

More interesting than the conserved backbone hydrogen bonds are the non-conserved interactions involving sidechains from the C-terminal ends of the two peptides. These C-terminal amino acids have large contact areas and buried surfaces, correlating with these residues contributing most strongly to the peptide binding affinities, as measured by competitive fluorescence polarization titrations involving SMRT or BCOR peptides that have single amino acid substitutions to either alanine for non-alanine residues or glycine for alanine residues [34]. To be considered useful, the SILCS method should be capable of recapitulating these important interactions.

The SILCS FragMaps capture every one of the thermodynamically important C-terminal sidechain interactions of the SMRT peptide with BCL-6. In the SMRT peptide, Arg1423, Ser1424, Ile1425, Asp1427, Ile1428, and Pro1429 in the C-terminal half make large contributions to the binding affinity [34]. Analysis of the crystal structures shows that the sidechains of Arg1423, Ser1424, and Asp1427 all form hydrogen bonds to BCL-6, while both the Ile1425 and Ile1428 aliphatic sidechains are buried in hydrophobic pockets. High-probability regions in the hydrogen-bond donor FragMap overlap with the polar hydrogens in the Arg1423 and Ser1424 sidechains, and high-probability regions in the hydrogen-bond acceptor FragMap overlap with the oxygens in the Ser1424 and Asp1427 sidechains (Figure 5*A*). High-probability regions in the aliphatic carbon FragMap encompass both the Ile1425 and Ile1428 sidechains (Figure 5*A*). Interestingly, only the Ile1428 sidechain and not the Ile1425 sidechain is also overlapped by a high-density region in the aromatic carbon FragMap. The lack of observable aromatic carbon FragMap density coincident with the Ile1425 sidechain occurs on both sides of the BCL-6 protein, and decreasing the isovalue threshold by half continues to yield no observable density on one side and only two small points of observable density on the other side that are overwhelmingly enveloped by the aliphatic carbon FragMap contour. This suggests that the Ile1428 pocket can accommodate both aliphatic and aromatic carbons, while the Ile1425 pocket will preferentially bind aliphatic carbons. Experimental evidence to this effect exists in the form of a crystal structure of BCL-6 with a small-molecule inhibitor, in which an aromatic moiety binds in the Ile1428 pocket (G. Privé, personal communication). Such differentiation emphasizes the ability of the SILCS method to account for the subtle energetic contributions that dictate the binding of different classes of hydrophobic moieties.

Pro1429 is interesting in that it is the only amino acid in the C-terminal region of the SMRT peptide that makes a large experimental thermodynamic contribution to binding yet whose sidechain is not involved in an interaction with the BCL-6 protein. Rather, its backbone carbonyl acts as a hydrogen bond acceptor, and this interaction is indeed seen in the corresponding FragMap (Figures 4*C* and 5*A*). This result indicates that Pro1429Ala mutation likely has a strong affect on the SMRT binding affinity due to an increase in conformational entropy and the fact that proline occupies the extended region of φ/ψ space while alanine preferentially occupies the helical region [39].

The SILCS FragMaps also capture the thermodynamically important interactions of the BCOR peptide C-terminal residues 508–512 (Figure 5*B*). These include sidechain interactions for Ser508, Trp509 and Val511. Surprisingly, no overlap is seen for the Val510 sidechain with a high-density region in either the aliphatic or aromatic FragMaps. This may have an explanation similar to that for SMRT Pro1429, in that the Val510Ala mutation may cause a decrease in binding affinity due to replacement of an amino acid that prefers an extended conformation with the helix-promoting alanine. Finally, as with the homologous SMRT Pro1429, the BCOR Pro512 backbone overlaps with a high-density region in the hydrogen bond acceptor FragMap while no sidechain overlap is seen (Figures 4*C* and 5*B*).

### SILCS captures protein flexibility

Because SILCS uses all-atom explicit-solvent MD simulations, protein flexibility is naturally included. As observed crystallographically, there are important differences in the conformations of BCL-6 sidechains in the peptide-binding groove between the BCL6 apo, BCL-6∶SMRT and BCL-6∶BCOR crystal structures. For example, BCL-6 Arg24 sidechain dihedral angles have significantly different values in crystal structures of the unliganded protein, the BCL-6∶SMRT complex, and the BCL-6∶BCOR complex, while the BCL-6 His116 sidechain undergoes a dramatic rearrangement between the SMRT and BCOR complexes. SILCS simulations seeded with a single BCL-6 structure capture this heterogeneity in both Arg24 (Figure S4) and His116, and can therefore inform the design of inhibitors targeting such flexible binding sites.

**Figure 6:**
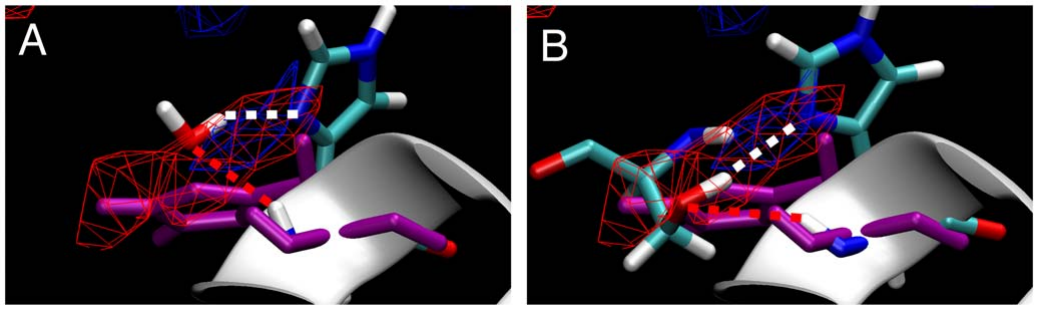
Conformations of BCL-6 His116 in the BCL-6∶SMRT cocrystal, sampled during SILCS MD, and in the BCL-6∶BCOR cocrystal. (A) The BCL-6∶SMRT cocrystal His116 conformation used to seed the simulations (purple) overlaid with a SILCS MD snapshot of the His116 conformation having an interacting water molecule (colored by atom type). (B) The BCL-6∶SMRT cocrystal His116 conformation (purple) overlaid with the BCL-6∶BCOR cocrystal His116 conformation and BCOR Ser508 (colored by atom type). The hydrogen bond donor FragMap is shown as a blue isocontour mesh, the hydrogen bond acceptor FragMap as a red isocontour mesh, and the helix containing His116 is represented as a white ribbon.

The SILCS MD behavior of the BCL-6 His116 sidechain is especially relevant because of the large crystallographically-determined conformational change required in this sidechain for BCL-6 to accommodate both the SMRT and the BCOR peptides. SILCS MD samples both the His116 sidechain conformation observed in the BCL-6∶SMRT crystal structure used to initiate all the SILCS simulations, and the very different conformation in the BCL-6∶BCOR crystal structure (Figure 6). In the SILCS MD, the His116 sidechain reversibly shifts between the initial, BCL-6∶SMRT conformation (Figure 6*A*, purple) and a second conformation. In this second conformation, His116 forms a hydrogen-bonding complex with a water molecule that acts as a hydrogen bond donor to the sidechain and as an acceptor to the His116 backbone amide NH group (Figure 6*A*, colored by atom type), a complex not possible in the initial conformation due to the location of the sidechain. Furthermore, this MD second conformation is the same as in the BCL-6∶BCOR crystal structure and enables hydrogen bonding between BCL-6 His116 and the BCOR Ser508 sidechain hydroxyl in the BCL-6∶BCOR crystal structure (Figure 6*B*). The Ser508 hydroxyl donates a hydrogen bond to the His116 sidechain and accepts a hydrogen bond from the His116 backbone amide NH group (Figure 6*B*) in the same manner as the water molecule in the simulation (Figure 6*A*). Importantly, the hydrogen bond donor and acceptor FragMaps show that these are high-probability (favorable free energy) interactions. These results demonstrate the ability of SILCS to include protein flexibility and the ability of the method to identify locations of favorable interaction sites on the protein surface that arise from protein flexibility. The conformational changes that SILCS can take into account are, naturally, related to the timescales of the MD simulations and of the conformational changes themselves. The present results suggest that readily-accessible timescales can account for the conformational heterogeneity in biologically important surface-exposed sidechains, although in situations with, for example, strong sidechain hydrogen bonding or large structural changes like loop opening, this may not be the case.

### Conclusions

Described is a new computational method that combines ideas from experimental fragment-based drug discovery with all-atom explicit-solvent molecular dynamics. The SILCS (Site Identification by Ligand Competitive Saturation) method, by using all-atom explicit solvent molecular dynamics, incorporates atomic-level solvation effects and protein mobility. The resulting 3D free energy-based probability distributions (FragMaps) suggest the optimal placement of aliphatic hydrophobic, aromatic, hydrogen-bond donor, and hydrogen-bond acceptor functionalities in a binding pocket. As an example, SILCS FragMaps computed for the BCL-6 oncoprotein do an excellent job of reproducing the binding interactions of the non-homologous SMRT and BCOR peptides with the BCL-6 protein and include biologically relevant conformational changes in the binding pocket.

SILCS FragMaps, when visualized as isosurfaces in conjunction with a protein (e.g. Figures 4–6), may potentially be used to guide the development of inhibitors at a particular site on the protein surface. The FragMaps contain information about protein flexibility and atomically-detailed solvation effects as they impact fragment binding. Additionally, the relative importance of interactions is represented by the values of the histogram counts in the 3D FragMap histograms, thus inhibitors can be optimally designed by targeting overlap with high-probability regions in the FragMaps. This can be done in an interactive, qualitative fashion, for example by informing the extension of small-molecule binders with known binding poses into larger, higher-affinity molecules that encompass nearby high-probability regions. Alternatively, this can be done in an automated, quantitative manner by taking the natural logarithm of the probabilities and multiplying by –*RT*; the resultant free-energy maps can be used as docking grids for high-throughput *in silico* docking of drug-like compound libraries, with an additional map of the protein atoms incorporated into a penalty function to account for steric clash between docked compounds and the protein. With this latter approach, some care must be taken regarding the direct interpretation of FragMaps in terms of free energies due to alterations to the chemical potential of bulk water, which is used to generate hydrogen bond donor and acceptor maps, arising from the high concentration of fragments. Additionally, some care may be required to delineate mutually-exclusive high-probability regions arising from protein conformational heterogeneity. Nonetheless, the use of SILCS free-energy FragMaps as docking grids has the potential to be a significant improvement over current high-throughput *in silico* methods, which are limited in their descriptions of protein flexibility and solvation [13].

Finally, an important part of the SILCS method is its computational feasibility. Each 5-ns SILCS simulation of BCL-6 took less than three days on a single 2×4-core node of a commodity computing cluster, and because each of the ten simulations was independent, they were all run simultaneously to yield converged FragMaps in under three days. The ability to achieve converged FragMaps probability maps in such a short time is a very important result, since MD simulations are often limited by the computational cost for simulations beyond the nanosecond regime, which in turn limits their utility in computer-aided drug discovery [13].

## Methods

### SILCS MD simulations

The experimental BCL-6 protein conformation from the BCL-6∶SMRT complex [33] [PDB ID 1R2B] was used to seed all SILCS MD simulations. The Reduce software [40] was used to place missing hydrogen positions and to choose optimal Asn and Gln sidechain amide and His sidechain ring orientations. Propane and benzene molecules were placed on a square grid, with the identity of the molecule at each grid point randomly determined. Ten such grids were generated with the grid spacing selected to yield a concentration of ∼1 M propane and ∼1 M benzene when combined with a box of water molecules at the experimental density of water. Ten protein+small molecule+water systems were generated by overlaying the coordinates of the BCL-6 protein and water molecules from the BCL-6∶SMRT co-crystal structure with each of the ten different solutions, removing all water, propane, and benzene molecules that overlapped the protein, and replacing two random water molecules with chloride ions to give a net neutral system charge. The final systems were rectangular boxes of size 72×58×43 Å to accommodate the protein with maximum dimensions of 64×48×35 Å.

Harmonic positional restraints with a force constant of 1 kcal*mol^−1^*Å^−2^ were placed on all protein atoms and the system was minimized for 500 steps with the steepest descent algorithm [41] under periodic boundary conditions [37]. Molecular dynamics simulations were performed on each minimized system using the “leap frog” version of the Verlet integrator [37] with a 2-fs timestep to propagate the system. The SHAKE algorithm [42] was applied to constrain bonds to hydrogen atoms to their equilibrium lengths and maintain rigid water geometries, long-range electrostatic interactions were handled with the particle-mesh Ewald method [43] with a real-space cutoff of 8 Å, a switching function [38] was applied to Lennard-Jones interactions in the range of 5 to 8 Å, and a long-range isotropic correction [37] was applied to the pressure for Lennard-Jones interactions beyond the 8 Å cutoff length. With the positional restraints still in place, the system was heated to 298 K over 20 ps by periodic reassignment of velocities [44], followed by 20 ps of equilibration at 298 K, also using velocity reassignment. After the heating and equilibration periods, the positional restraints were replaced by restraints on only protein backbone C_α_ positions with a very weak force constant of 0.01 kcal*mol^−1^*Å^−2^ so as to prevent rotation of the protein in the rectangular simulation box. Each system was subsequently simulated for 5 ns at 298 K and 1 atm, with the Nosé-Hoover thermostat [45],[46] and the Langevin piston barostat [47], for a total of 50 ns of simulation time. All simulations were done with the CHARMM molecular simulation software [48], the CHARMM protein force field [49] with CMAP backbone correction [50], and the TIP3P water model [51] modified for the CHARMM force field [52].

### FragMap construction

FragMaps were prepared for each SILCS simulation by binning atoms from SILCS MD snapshots taken at 2-ps intervals into 1 Å×1 Å×1 Å cubic volume elements of a grid spanning the entire system. For the aliphatic and aromatic carbon FragMaps, carbon atoms for propane and benzene molecules, respectively, were binned if they were within 5 Å of the protein. For the hydrogen bond donor and acceptor FragMaps, water hydrogen and oxygen atoms, respectively, were binned if they were within 2.5 Å of the protein. For each type of FragMap, the respective FragMaps from each of the ten simulations were added together to create a single FragMap. A single isocontour value resulting in optimal visualization was empirically chosen for each FragMap type, and this value was used to generate all isocontour molecular graphics for that FragMap type. The ratio of the isocontour value to the average cubic volume element occupancy in an equilibrated system consisting of only propane, benzene, and water molecules was 9.8 for propane carbons, 9.8 for benzene carbons, 1.3 for water hydrogens, and 1.1 for water oxygens. Visualization of FragMaps and preparation of molecular graphics were done with VMD [53].

## Supporting Information

Figure S1 Successive slices of the aromatic carbon atom FragMap, generated by mapping benzene carbon atoms, and the BCL-6 molecular surface taken perpendicular to the two-fold symmetry axis of the protein. A through D are the same slices as in Figure 2. E is an additional successive slice.
(1.44 MB TIF)

Figure S2 Successive slices of the hydrogen bond donor FragMap, generated by mapping water hydrogen atoms, and the BCL-6 molecular surface taken perpendicular to the two-fold symmetry axis of the protein. A through D are the same slices as in Figure 2. E is an additional successive slice.
(1.49 MB TIF)

Figure S3 Successive slices of the hydrogen bond acceptor FragMap, generated by mapping water oxygen atoms, and the BCL-6 molecular surface taken perpendicular to the two-fold symmetry axis of the protein. A through D are the same slices as in Figure 2. E is an additional successive slice.
(1.52 MB TIF)

Figure S4 Arg24 sidechain (A) χ1, (B) χ2, (C) χ3, and (D) χ4 dihedral distributions from the SILCS MD simulations. The starting dihedral values from BCL-6 in the BCL-6∶SMRT complex [PDB ID 1R2B] are shown as solid lines, and the dihedral values in unliganded BCL-6 [PDB ID 1R28, 1R29] and in the BCL-6∶BCOR [PDB ID 3BIM] complex are shown as dashed lines.
(0.73 MB TIF)
