# Supplementary figures and images for: Computational Fragment-Based Binding Site Identification by Ligand Competitive Saturation
Source: PLoS Comput Biol. 2009 Jul 10;5(7):e1000435. doi: 10.1371/journal.pcbi.1000435 (PMC2700966; doi:10.1371/journal.pcbi.1000435)

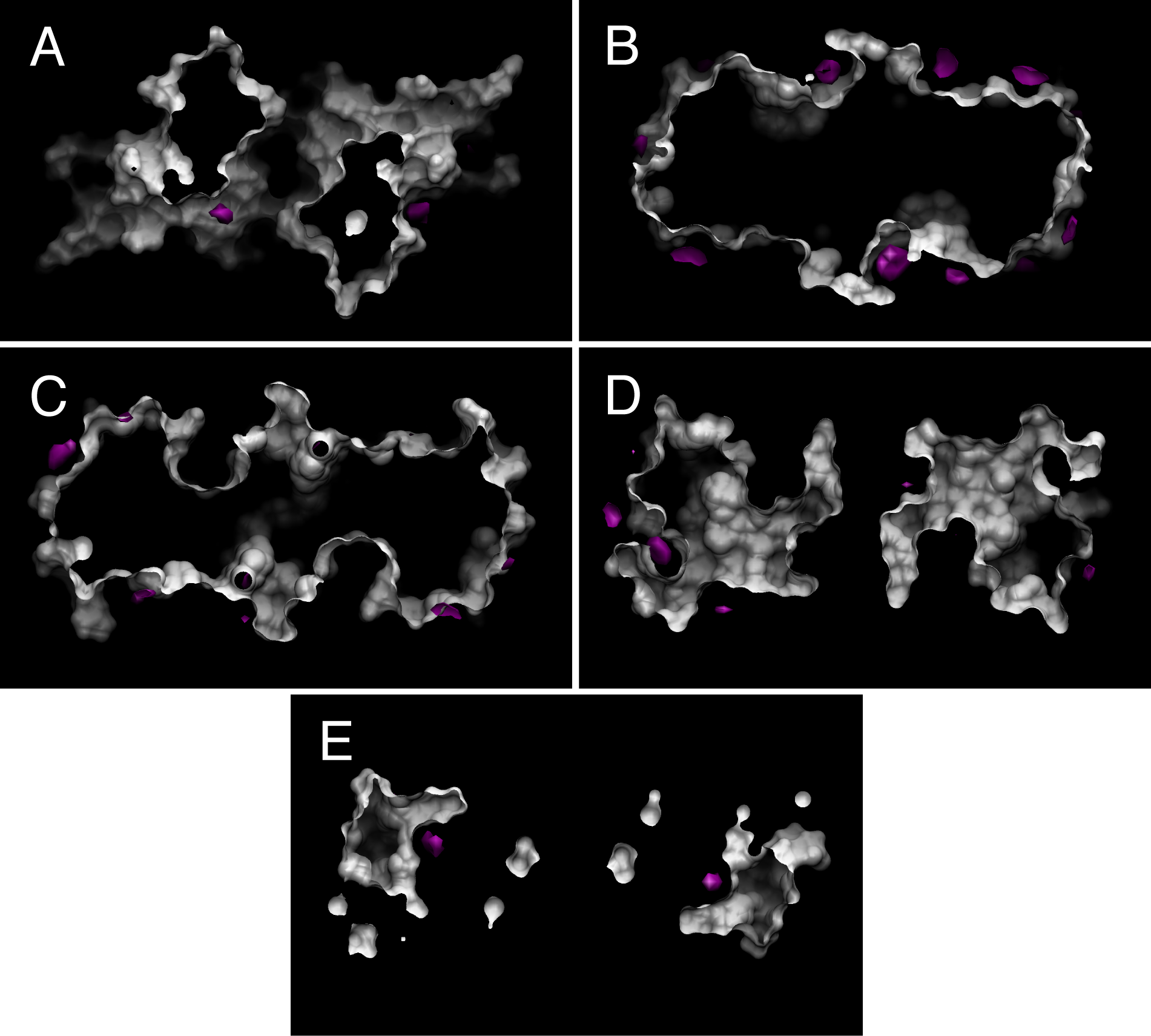

Supplement: Figure S1 — Successive slices of the aromatic carbon atom FragMap, generated by mapping benzene carbon atoms, and the BCL-6 molecular surface taken perpendicular to the two-fold symmetry axis of the protein. A through D are the same slices as in Figure 2. E is an additional successive slice. (1.44 MB TIF) [file pcbi.1000435.s001.tif]

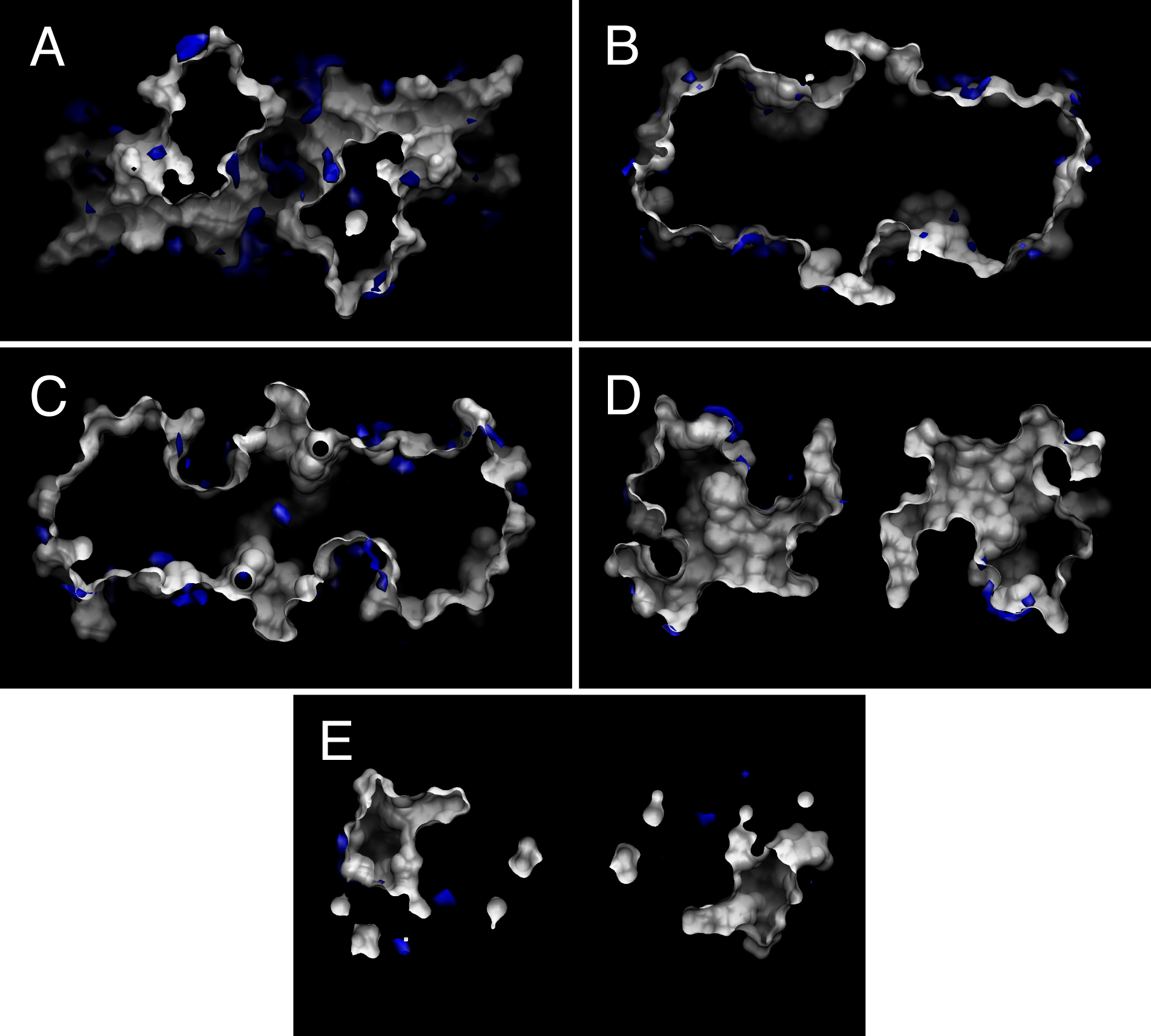

Supplement: Figure S2 — Successive slices of the hydrogen bond donor FragMap, generated by mapping water hydrogen atoms, and the BCL-6 molecular surface taken perpendicular to the two-fold symmetry axis of the protein. A through D are the same slices as in Figure 2. E is an additional successive slice. (1.49 MB TIF) [file pcbi.1000435.s002.tif]

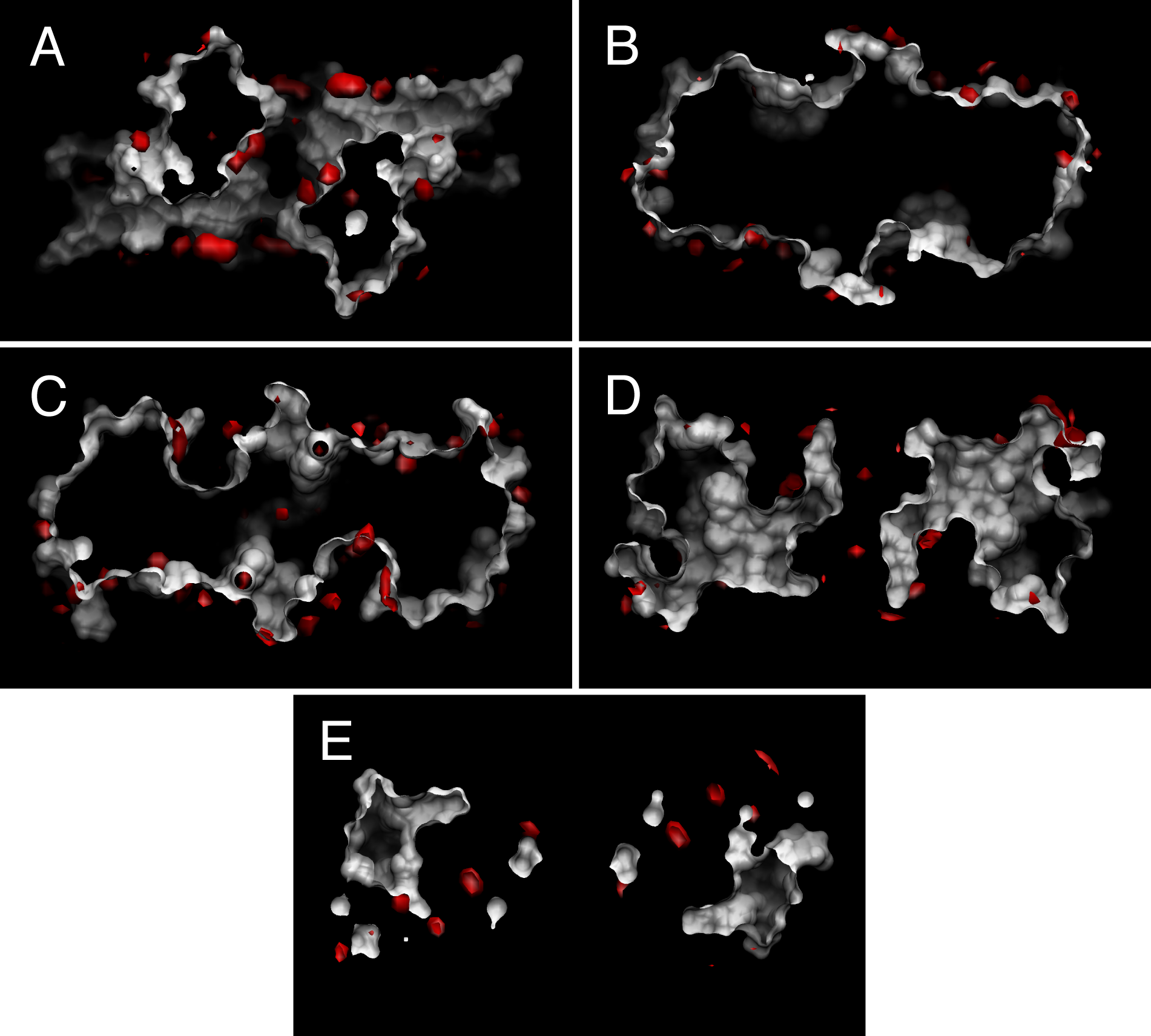

Supplement: Figure S3 — Successive slices of the hydrogen bond acceptor FragMap, generated by mapping water oxygen atoms, and the BCL-6 molecular surface taken perpendicular to the two-fold symmetry axis of the protein. A through D are the same slices as in Figure 2. E is an additional successive slice. (1.52 MB TIF) [file pcbi.1000435.s003.tif]

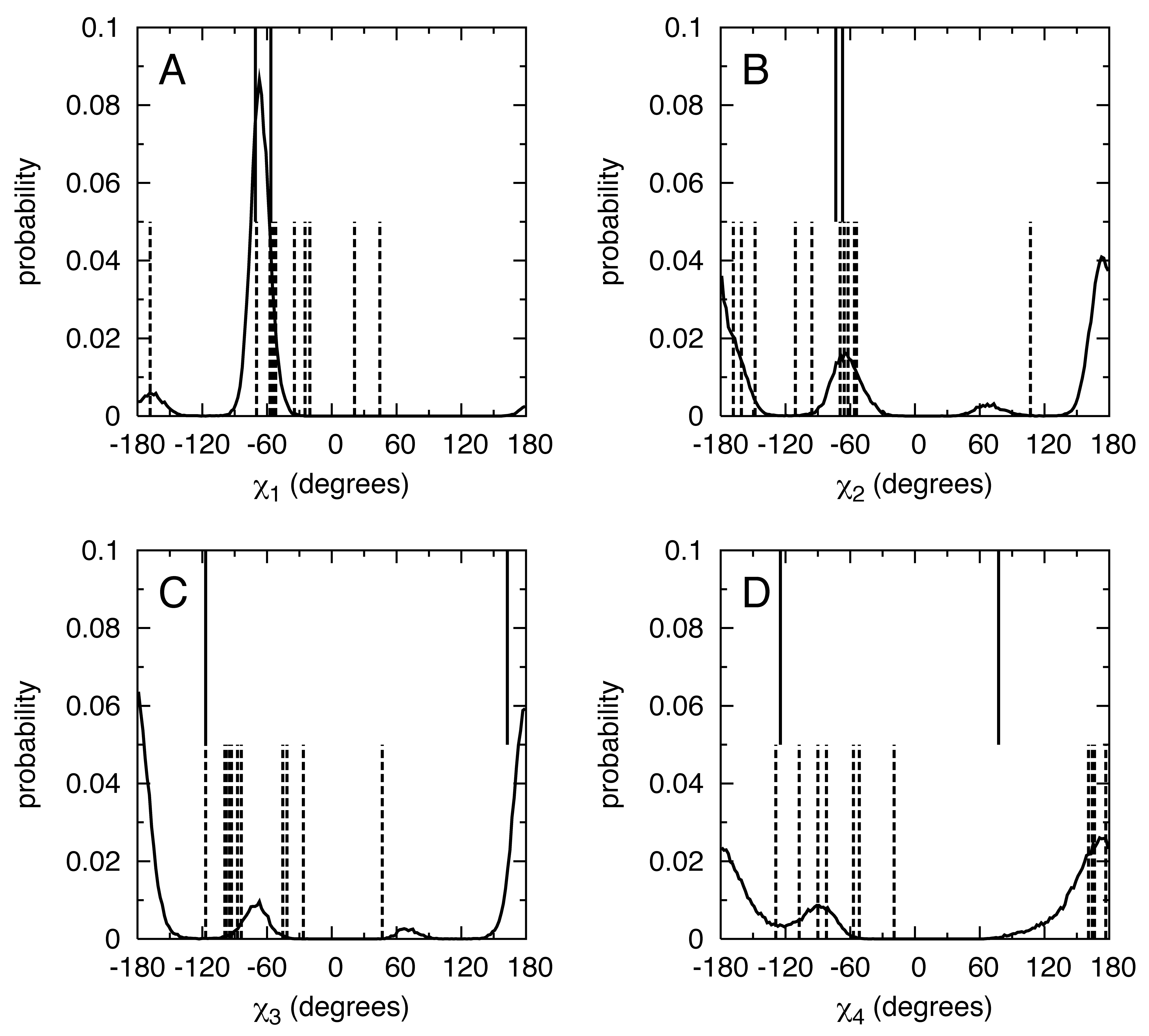

Supplement: Figure S4 — Arg24 sidechain (A) χ1, (B) χ2, (C) χ3, and (D) χ4 dihedral distributions from the SILCS MD simulations. The starting dihedral values from BCL-6 in the BCL-6∶SMRT complex [PDB ID 1R2B] are shown as solid lines, and the dihedral values in unliganded BCL-6 [PDB ID 1R28, 1R29] and in the BCL-6∶BCOR [PDB ID 3BIM] complex are shown as dashed lines. (0.73 MB TIF) [file pcbi.1000435.s004.tif]
